# Suppression of low-density lipoprotein oxidation, vascular smooth muscle cell proliferation and migration by a herbal extract of *Radix Astragali, Radix Codonopsis *and *Cortex Lycii*

**DOI:** 10.1186/1472-6882-11-32

**Published:** 2011-04-22

**Authors:** Judy Y Chan, Johnny C Koon, Ping-Chung Leung, Chun-Tao Che, Kwok-Pui Fung

**Affiliations:** 1 Institute of Chinese Medicine, The Chinese University of Hong Kong, Shatin, N.T., Hong Kong SAR, China; 2 State Key Laboratory of Phytochemistry and Plant Resources in West China, The Chinese University of Hong Kong, Shatin, N.T., Hong Kong SAR, China; 3 School of Biomedical Sciences, The Chinese University of Hong Kong, Shatin, N.T., Hong Kong SAR, China; 4 School of Chinese Medicine, The Chinese University of Hong Kong, Shatin, N.T., Hong Kong SAR, China

**Keywords:** Atherosclerosis, vascular smooth muscle cell, proliferation, migration

## Abstract

**Background:**

Atherosclerosis is a major cause of death in developed world. Atherosclerosis is characterized by low-density lipoprotein deposition in the arterial wall which ultimately begets the formation of lesions. Rupture of lesions finally leads to clinical events such as heart attack and stroke. Atherosclerosis is a complication associated with diabetes. In patients with diabetes, the risk of atherosclerosis is three to five folds greater than in non-diabetics. Our previous study showed that a herbal extract of *Radix Astragali, Radix Codonopsis *and *Cortex Lycii*, namely SR10, could improve glucose homeostasis both *in vitro *and *in vivo*. In this study, we want to further investigate the efficacy of SR10 in treating atherosclerosis.

**Method:**

The inhibitory effect of SR10 on low-density lipoprotein oxidation was investigated using free radical-induced erythrocyte hemolysis model and copper ion-induced low-density lipoprotein oxidation model. Since vascular smooth muscle cell proliferation and migration are important processes in atherogenesis, we also examined the effect of SR10 in inhibiting these events.

**Results:**

Our results showed that SR10 inhibited erythrocyte hemolysis with IC_50 _value at 0.25 mg/ml and significantly prolonged low-density lipoprotein oxidation *in vitro*. SR10 attenuated platelet derived growth factor-BB-induced vascular smooth muscle cell proliferation by promoting cell cycle arrest at G_0_/G_1 _phase as well as inhibiting vascular smooth muscle cell migration.

**Conclusion:**

The potential application of SR10 in treating atherosclerosis has been implied in this study. Animal model will be needed to further verify the efficacy of SR10 in future.

## Background

Diabetes mellitus (DM) affects more than 170 million people in the world. Due to associated complications, the mortality rate in DM patients is much higher than that in non-DM patients. Of various diabetic complications, atherosclerosis represents the major mortal threat to DM patients [1,2]. Many studies have been carried out to find the association between diabetes and atherosclerosis.

It was found that glucose-enhanced low density lipoprotein (LDL) oxidation and glucose-mediated enhancement of LDL oxidation was partially blocked by superoxide dismutase [3,4].

Oxidized LDL is involved in atherogenesis by affecting cytokine production, endothelium-derived relaxing factor-mediated vascular reactivity and foam cell formation [5,6].

These findings explain how chronic hyperglycemia of diabetes accelerates lipoprotein oxidation, thereby promoting diabetic vascular disease.

It was found that free radicals are one of the major causes of atherogenesis. Oxygen derived free radicals are very important mediators of cell injury. These free radicals include superoxide, hydrogen peroxide and nitric oxide. Collectively, the high activity of reactive oxygen species (ROS) determines chemical changes in virtually all cellular components, leading to DNA and protein modification and lipid peroxidation [7]. In addition, excessive ROS in diabetes is thought to promote atherogenesis by affecting several steps. Firstly, it facilitates monocyte and macrophage recruitment. Secondly, it increases lipid deposition in the intimal layer. Thirdly, it promotes the proliferation and migration of smooth muscle cells [8]. One of the principal regulators of mitogenesis in vascular smooth muscle cells is platelet-derived growth factor-BB (PDGF-BB). The signaling pathway of PDGF-BB-induced mitogenesis involves the activation of extracellular regulated kinases 1 and 2 (ERK1/2) [9]. ERK1/2-mediated pathway was also shown to be important for PDGF-BB-induced cell cycle progression in vascular smooth muscle cells. Within the arterial media, smooth muscle cells are mostly in the quiescent stage (i.e. G_0_/G_1 _phase of the cell cycle). Upon vessel injury, smooth muscle cells migrate into the intima, where they transit through G_1 _phase and enter the S phase.

In our previous study, a herbal mixture, namely SR10, comprising the aqueous extracts of *Radix Astragali, Radix Codonopsis *and *Cortex Lycii *was examined for its anti-diabetic and anti-oxidative effects both *in vitro *and *in vivo *[10,11]. In the diabetic mouse model, the activities and expression of the anti-oxidant enzymes, catalase and superoxide dismutase, were up-regulated when treated with SR10. This anti-oxidative property implied the therapeutic potential of SR10 in treating atherosclerosis. Therefore, in this study, we investigated the effect on oxidative resistance of LDL which is an important step in initiating atherosclerosis. Furthermore, the effect of SR10 on PDGF-BB-induced vascular smooth muscle cell proliferation and migration was examined.

## Methods

### Sources and authentication of herbal materials

*Radix Astragali*, dried root of Astragalus membranaceus (Fisch.) Bge., *Radix Codonopsis*, dried root of Codonopsis pilosula (Franch.) Nannf. and *Cortex Lycii*, dried root bark of Lycium chinense Mill., were purchased from a herbal pharmaceutical company in Hong Kong. All herbs were authenticated by morphological observation and thin layer chromatography (TLC) according to the method described in Pharmacopoeia of the People's Republic of China 2000 [12]. The reference compounds and reference herbs used in authentication were purchased from National Institute for the Control of Pharmaceutical and Biological Products in China. The authenticated voucher specimen were deposited in the Institute of Chinese Medicine, The Chinese University of Hong Kong with voucher numbers (*Radix Astragali*, 2005-2580; *Radix Codonopsis*, 2005-2597; *Cortex Lycii*, 2005-2601).

### Preparation of herbal extracts

SR10 was prepared as described previously by boiling 214.3 g of *Radix Astragali*, 214.3 g of *Radix Codonopsis *and 71.4 g of *Cortex Lycii *in 5 L of distilled water for 2 hours under reflux [10] and collecting the extract. Another 5 L of distilled water was added and the boiling process was repeated. Two batches of water extract were mixed together and centrifuged to remove the herbal debris. The extract was vacuum dried and the resulting herbal powder was stored at -20°C until use. The powder contained 25.5 g/100 g of the starting raw material.

### Cell Culture

A7r5, a rat aorta smooth muscle cell line, was purchased from American Type Culture Collection (ATCC number CRL-1444) and maintained in RPMI-1640 medium supplemented with 10% fetal bovine serum and 1% penicillin-streptomycin in a humidified atmosphere of 5% CO_2 _at 37°C.

### Measurement of free radical-induced erythrocyte hemolysis

Blood was collected from adult Sprague-Dawley (SD) rat from thoracic aorta by heparinised tube. Red blood cells were obtained by centrifugation at 1500 × g for 10 minutes and washed twice with 0.15 M NaCl solution. After centrifugation, 20% RBC suspension was obtained by resuspending RBC in four times the volume of 0.15 M NaCl solution. RBC lysis reaction was set up in microcentrifuge tubes, each containing 10% RBC suspension, 100 mM of 2,2'-azo-bis-(2-amidinopropane) dihydrochloride (AAPH) and ascorbic acid (positive control) or various concentrations of SR10 in a total volume of 1 ml. Control was set up using PBS instead of SR10. RBC with ascorbic acid or SR10 was added first, and the reaction initiated by adding 100 mM of AAPH. The mixtures were then incubated in an oscillator at 37°C for 200 minutes. After incubation, the mixtures were diluted with PBS or distilled water by 20-fold respectively. The diluted mixtures were centrifuged at 1500 × g for 10 minutes. The supernatant (200 μl) of each mixture was transferred to a 96-well microtiter plate for measurement at 540 nm by microplate reader. Percentage inhibition of RBC hemolysis was calculated by the equation: Inhibition % = (A - B) × 100%, whereas A = (DW_sample _- PBS_sample_)/DW_sample_; B = (DW_control _- PBS_control_)/DW_control_

### Measurement of LDL peroxidation

The reaction was set up in a quartz cuvette, each containing 75 μg of LDL, 5 μM of copper (II) chloride and various concentrations of SR10 in a total volume of 1 ml. LDL and the testing drug were added to the cuvette before adding copper (II) chloride to initiate the reaction. Conjugated dienes formation was continually monitored at 37°C by measuring UV absorption at 234 nm in 5-minute intervals for a total of 24 hours. The lag time (L.T.) for the formation of conjugated dienes was determined to be the intercept of the slopes for the lag and propagation phases, and was compared to the control (using PBS instead of SR10).

### Cell proliferation assay

A7r5 cells (0.5 × 10^4^/well) were seeded in each well of 96-well culture plate. After overnight incubation, PDGF-BB (25 ng/ml) was added to the cells in the presence or absence of various concentrations of SR10. Cells without the addition of PDGF-BB and SR10 was used as negative control. After further incubation for 24 hours, 3-(4,5-dimethyl-thiazol-2-yl)-2,5-diphenyltetrazolium (MTT) assay was performed to measure cell viability [11]. Briefly, medium was removed and 40 μl of MTT solution (5 mg/ml in PBS) was added to each well. After incubation for 4 hours at 37°C, MTT solution was removed and 100 μl of dimethyl sulfoxide was added to dissolve the crystals formed. Then, absorbance at 540 nm was read using a microplate reader. The percentage cell viability was calculated as [Absorbance_(treatment)_/Absorbance_(negative control)_] × 100%.

### Determination of DNA synthesis

DNA synthesis in A7r5 cells was determined by ^3^H-thymidine uptake assay. Cells (2 × 10^3^/well) were seeded in a 96-well plate and incubated overnight. The cells were synchronized by starving in 1% fetal bovine serum for another 24 hours. PDGF-BB was added in the presence or absence of various concentrations of SR10 and further incubated at 37°C with 5% CO_2 _for 24 hours. Subsequently, tritiated thymidine (0.5 μCi per well) was added into each well and incubated for 6 hours. After that, the cells were harvested on glass fiber filters by a cell harvester. Radioactivity in the filters was measured by Microplate Scintillation and Luminescence Counter (Topcount NXT™).

### Cell cycle analysis by PI staining using flow cytometer

Cells (2 × 10^5^/well) were seeded in a 6-well plate and incubated overnight. The cells were synchronized by starving in 1% fetal bovine serum for another 24 hours. PDGF-BB was then added in the presence or absence of various concentrations of SR10 and further incubated at 37°C with 5% CO_2 _for 24 hours. Cell cycle analysis was performed by PI staining using flow cytometry as described previously [13]. In brief, the cells were harvested, washed twice with PBS and fixed overnight with 70% ethanol. After fixation, the cells were washed with PBS and resuspended in 400 μl of PBS, 50 μl of RNase A (10 mg/ml) and 10 μl of propidium iodide (PI, 2 mg/ml). The cells were further incubated at 37°C for 30 minutes before analysis by FACSort flow cytometry (Becton Dickinson) using 'Cell Quest' software. The cell population was chosen by forward scatter (FSC) light and side scatter (SSC) light. The signal was detected by FL3 channel for PI with log scale.

### Cell migration assay

Cell migration assay was performed in modified Boyden chambers using Transwell (Costar) culture chambers with membrane pore size of 8 μm. A7r5 cells (1.5 × 10^4^/well) in serum-free DMEM were loaded in the upper compartment (100 μl). PDGF-BB dissolved in plain DMEM was placed in the lower compartment (600 μl) in the presence or absence of various concentrations of SR10. The chamber was incubated for 3 hours at 37°C in a humidified atmosphere containing 5% CO_2_/95% air. Cells on the membrane were fixed in 1% paraformaldehyde and stained in hematoxylin. Non-migrated cells on the upper surface were scraped away gently. The number of migrated cells at the lower surface was determined under microscope. Five regions were counted per filter. Three chambers were used for each treatment and control group. The experiment was performed in triplicate.

### Western blot analysis of extracellular regulated kinases 1 and 2 (ERK1/2) and cyclin D1

Western blot was performed as described previously [14]. After appropriate treatment, the cells were lysed in buffer containing 0.02% Aprotinin, 2% SDS, 10% glycerol, 62.5 mM Tris-HCl, pH 6.8 and the protein concentration was determined using the bicinchonic acid protein assay. Samples with equal amount of protein (25 μg) were analysed by polyacrylamide gel electrophoresis. The proteins were then transferred to a polyvinylidene fluoride (PVDF) membrane. After blocking with 10% non-fat milk in PBS-T (PBS with 0.1% Tween-20), the membranes were incubated overnight with antibodies against ERK1/2 or cyclin D1 or β-actin at 4°C. The membranes were further incubated with horseradish peroxidase-conjugated secondary antibodies for 1 hour and the signal detected by enhanced chemiluminescence (ECL) detection reagents (GE Healthcare).

### Data analysis

All experimental results were presented as mean ± standard deviation (S.D.). The Mann-Whitney test was used for comparison between PDGF-BB-treated group and each SR10 treatment group. The test was two-sided with a significance level of 0.05.

## Results

### Inhibition of AAPH-induced RBC hemolysis

**Figure 1:**
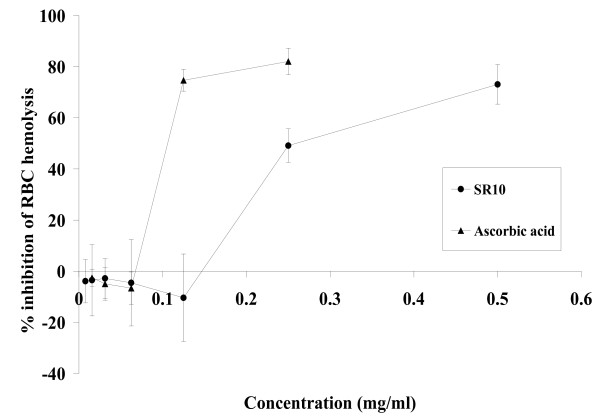
**Effect of SR10 on AAPH-induced hemolysis in erythrocytes**. Erythrocyte suspension was incubated with PBS (set as 0% inhibition), various concentrations of ascorbic acid (positive control) or SR10 in the presence of 100 mM AAPH for 200 minutes at 37°C. Values are expressed as mean ± S.D. of three independent experiments.

2,2'-azo-bis-(2-amidinopropane) dihydrochloride (AAPH) is a well-known free radical generator. In the absence of AAPH, hemolysis of RBC was negligible. When RBCs were incubated with 100 mM AAPH for 200 minutes, about 93% of hemolysis was detected (data not shown). However, percentage inhibition of hemolysis was increased when RBCs were incubated with increasing concentrations of SR10. In Figure 1, SR10 was shown to inhibit up to 70% hemolysis at concentration 1 mg/ml, with IC_50 _value at 0.25 mg/ml. Ascorbic acid (vitamin C) was used as a positive control with IC_50 _value at 0.1 mg/ml.

### Prolongation of LDL oxidation

**Figure 2:**
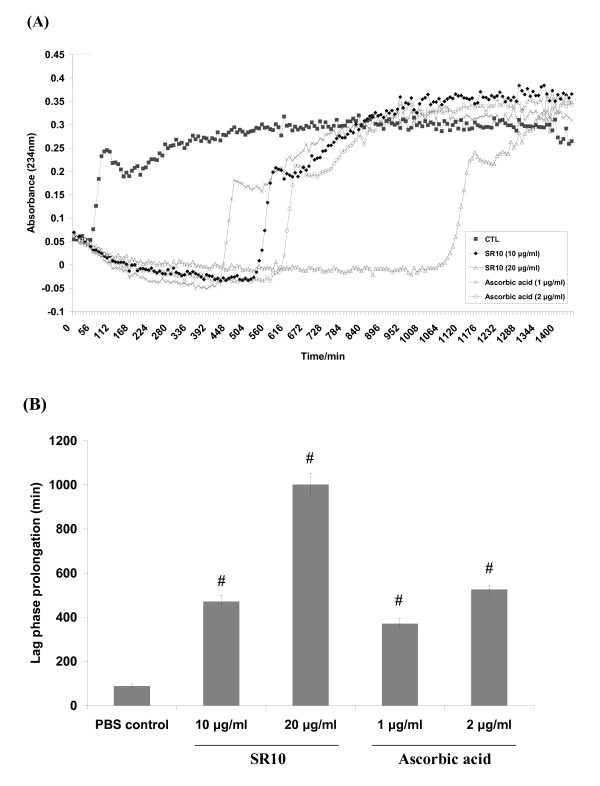
**Effect of SR10 on prolongation of copper ion-induced LDL oxidation**. LDL in PBS was incubated with 5 μM CuCl_2 _at 37°C in the presence or absence of SR10, and ascorbic acid (positive control). Conjugated diene formation was measured by determining the absorbance at 234 nm at every 5 minutes for totally 24 hours. A figure showing one representative experiment of three trials was shown in panel A. Lag time was determined as the intercept of the slopes for the lag phase and propagation phase. Difference of lag time between treatment and control (PBS only) was defined as lag phase prolongation. Results of lag phase prolongation time from three independent trials was shown in panel B. Values are expressed as mean ± S.D. for three independent experiments. By Mann-Whitney test, significant difference when compared to PBS control was indicated by # p < 0.01.

Lag phase prolongation, as calculated by lag time_(sample) _- lag time_(control)_, was used to measure the antioxidant property of the sample to protect from LDL oxidation. The lag time was determined graphically as the x-intercept of the tangent to the propagation curve. Figure 2A showed the result of one of the representative trial. Data shown in Figure 2B was the average of three trials. SR10 increased the lag time from 85 minutes (PBS control) to 480 minutes and 1000 minutes at concentrations of 10 μg/ml and 20 μg/ml, respectively. Ascorbic acid, as a positive control, increased the lag time to 370 minutes and 525 minutes at concentrations 1 μg/ml and 2 μg/ml, respectively.

### Inhibition of vascular smooth muscle cell proliferation

**Figure 3:**
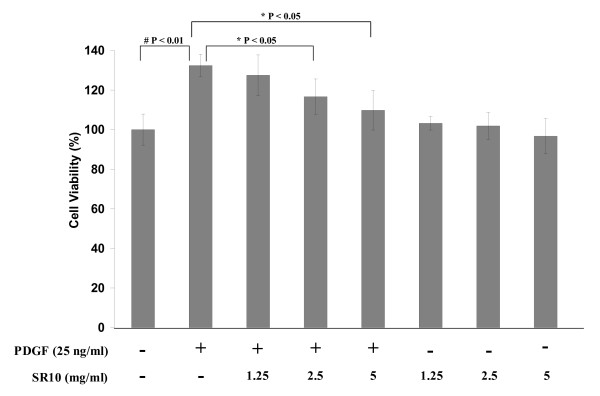
**Effect of SR10 on PDGF-BB-stimulated proliferation of A7r5 cells**. Cells were incubated with PDGF-BB (25 ng/ml) for 24 hours in the absence or presence of various concentrations of SR10. Cells incubated with various concentrations of SR10 without PDGF-BB were used to indicate the cytotoxicity of SR10. After 24 hours, MTT assay was performed to measure the cell viability in different treatments. Percentage of cell viability without treatment of PDGF-BB or SR10 was set as 100% (negative control). Percentage of cell viability of other treatment groups was calculated against negative control. Data are expressed as mean ± S.D. with eight replicates for each of three independent experiments. By Mann-Whitney test, significant difference when compared to PDGF-BB alone was indicated by *p < 0.05 or # p < 0.01.

The effect of SR10 on vascular smooth muscle cell proliferation was evaluated. A7r5 cells were incubated with PDGF-BB in the presence or absence of SR10 for 24 hours. Cell viability was examined by MTT assay. When A7r5 cells were stimulated with PDGF-BB for 24 hours in the absence of SR10, cell growth (which is directly proportional to absorbance measured in MTT assay) was significantly increased. However, the addition of SR10 suppressed this PDGF-BB-stimulated proliferation in a concentration-dependent manner with significant effect at concentrations of 2.5 mg/ml and 5 mg/ml. This inhibition of cell proliferation was not due to toxicity of SR10 as SR10 alone did not induce any significant change (Figure 3).

**Figure 4:**
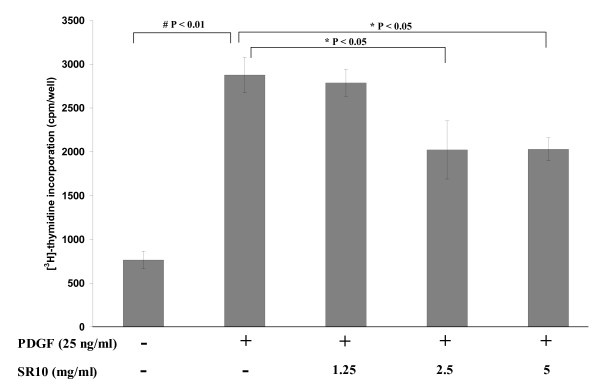
**Effect of SR10 on PDGF-BB-induced ^3^H-thymidine incorporation in A7r5 cells**. Cells were treated with PDGF-BB (25 ng/ml) for 24 hours in the absence or presence of SR10 and incorporation of ^3^H-thymidine was measured. Data was expressed as mean ± S.D. with six replicates for each of three independent experiments. By Mann-Whitney test, significant difference when compared to PDGF-BB alone was indicated by *p < 0.05 or # p < 0.01.

The effect of SR10 on cell growth was also detemined by measuring DNA synthesis. PDGF-BB highly increased ^3^H-thymidine incorporation into DNA but the increase was inhibited by co-treatment of SR10 in a concentration-dependent manner (Figure 4).

### Cell cycle arrest at G_0_/G_1 _and inhibition of expression of cyclin D1 and ERK1/2

**Figure 5:**
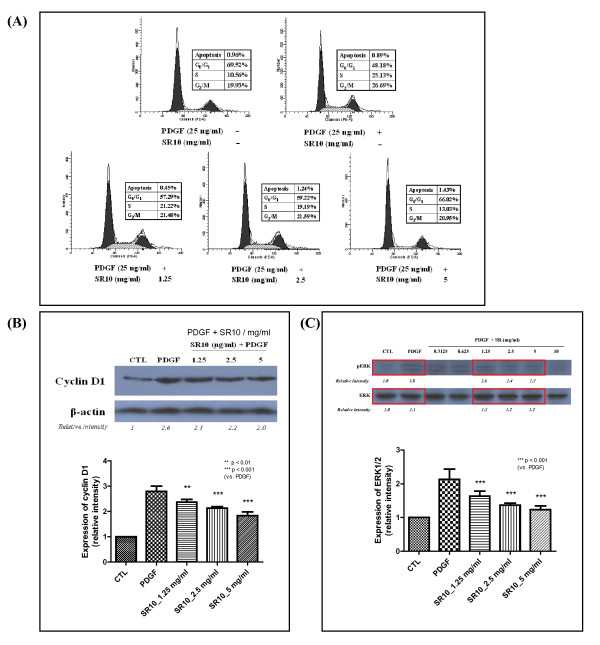
**Effect of SR10 on cell cycle distribution of PDGF-BB-treated A7r5 cells**. After starving with 1% fetal bovine serum for 24 hours, A7r5 cells were treated with PDGF-BB (25 ng/ml) in the absence or presence of SR10 for 24 hours. Then, the cells were stained with PI and cell cycle distribution was analyzed by flow cytometry. Three experiments were performed and similar results were observed. This figure showed the result of one representative experiment (A). The expression level of cyclin D1 was detected by Western blot. The figure is a representative of three independent experiments with similar results. The quantitation of cyclin D1 was performed by analyzing three sets of data by One-way ANOVA. Treatment of 1.25, 2.5 or 5 mg/ml of SR10 all suppressed the expression of cyclin D1 which was induced by PDGF. Statistical significance was indicated by ** p < 0.01 or *** p < 0.001 (B). The expression level of ERK1/2 was detected by Western blot. The figure is a representative of three independent experiments with similar results. The quantitation of ERK1/2 was performed by analyzing three sets of data by One-way ANOVA. Treatment of 1.25, 2.5 or 5 mg/ml of SR10 all suppressed the expression of cyclin D1 which was induced by PDGF (data shown in red boxes). Statistical significance was indicated by *** p < 0.001 (C).

The effect of SR10 on cell cycle progression was evaluated by PI staining. In quiescent A7r5 cells, the populations in G_0_/G_1_, S and G_2_/M phase were 69.52%, 10.56% and 19.93%, respectively. When the cells were stimulated by PDGF-BB for 24 hours, cell cycle progression from G_0_/G_1 _to S and G_2_/M phase was observed. However, treatment with various concentrations of SR10 blocked G_0_/G_1_-S phase transition. The population of G_0_/G_1 _phase was increased from 48.18% to 57.29%, 59.22% and 66.02% when PDGF-BB-stimulated cells were treated with SR10 at concentrations of 1.25 mg/ml, 2.5 mg/ml and 5 mg/ml, respectively. Populations in S phase were decreased from 25.13% to 21.22%, 19.19% and 13.03%, respectively, while in G_2_/M phase, the populations were decreased from 26.69% to 21.48%, 21.59% and 20.95%, respectively (Figure 5A).

Since SR10 arrested cell cycle progression at G_0_/G_1 _phase, G_1 _phase-regulated protein cyclin D1 expression was examined by Western blot. The results showed that SR10 mildly suppressed expression of cyclin D1 which was up-regulated by PDGF-BB (Figure 5B).

ERK1/2-mediated pathway is important for PDGF-BB-induced cell cycle progression in vascular smooth muscle cells. Western blot analysis showed that SR10 suppressed the proliferation of VSMC via the ERK pathway and phosphorylated ERK1/2 is one of the regulators affected (Figure 5C).

### Suppression of vascular smooth muscle cell migration

**Figure 6:**
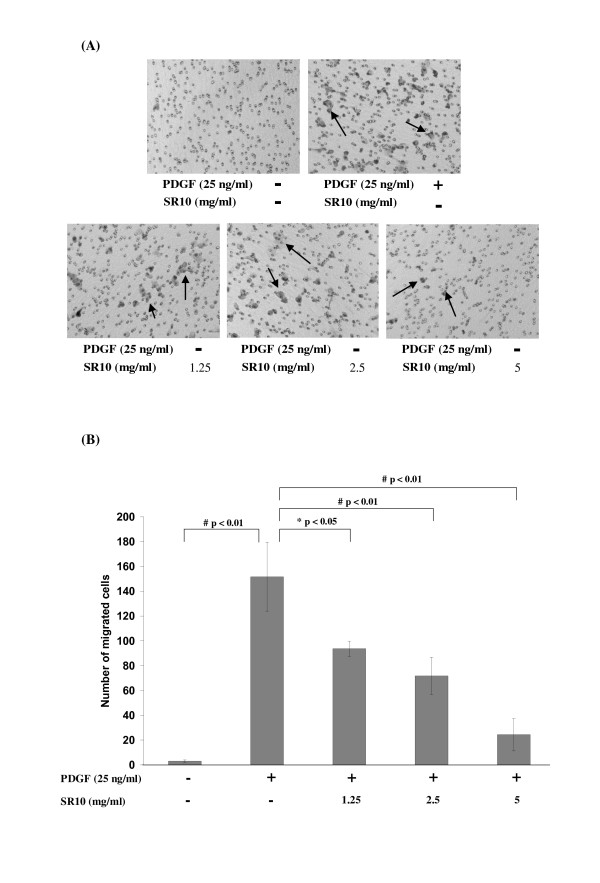
**Effect of SR10 on PDGF-BB-induced migration of A7r5 cells in a transwell migration assay**. A7r5 cells were loaded in the upper chamber while PDGF-BB (25 ng/ml) was loaded in the lower chamber in the absence or presence of various concentrations of SR10. After 3-hour incubation, migrated cells were observed in the lower surface of the membrane (A). Number of migrated cells was counted for five regions per filter. Data were expressed as mean ± S.D. with three replicates in each of three independent trials. By Mann-Whitney test, significant difference when compared to PDGF-BB alone was indicated by *p < 0.05 or # p < 0.01 (B).

Figure 6A shows the view of the lower membrane after cell migration through it. PDGF-BB greatly induced vascular smooth muscle cell migration. However, when SR10 was placed at the lower chamber with PDGF-BB, cell migration was inhibited significantly at all concentrations tested (Figure 6B).

## Discussion

Proliferation and migration of VSMCs are the basic pathological changes in atherosclerosis that lead ultimately to the formation of atherosclerotic plaque. Oxidized LDL (ox-LDL) has been shown to be participated in the initiation and development of atherosclerotic plaque by initiating foam cell formation, promoting the recruitment of circulating monocytes into the intima as well as activating the proliferation of vascular smooth muscle cells [15]. Oxidants for LDL oxidation include reactive oxygen species such as superoxide anion and peroxynitrite, as well as peroxidase and metal ions [16]. Since ox-LDL was formed by oxidation of LDL, anti-oxidant that can prevent this process may decrease the development of atherosclerosis. Thus, in this study, we measured the anti-oxidative activity of SR10 using AAPH-induced RBC hemolysis model and then assess the delay of LDL oxidation.

AAPH is a well-known free radical generator which induce lipid peroxidation on the RBC membrane. Our results demonstrated that SR10 inhibited AAPH-induced RBC hemolysis in a dose-dependent manner with IC_50 _found at 0.25 mg/ml. This showed that SR10 had an anti-oxidative property. We next examined the effect of SR10 on the oxidative resistance of LDL. Conjugated diene formation in LDL by copper ion-mediated oxidation was measured. In Figure 2, lag time is obtained from the graph to measure the ability to inhibit LDL oxidation. A longer lag time represents a stronger anti-oxidative activity to prolong LDL oxidation. Our results demonstrated that SR10 could inhibit LDL from being oxidized by copper ion. The results of both AAPH-induced and copper ion-induced lipid peroxidation indicated that SR10 is an anti-oxidant which could inhibit LDL oxidation.

Since LDL oxidation induces the initiation and development of atherosclerotic plaque, we next examined the effect of SR10 in proliferation and migration of VSMC which are important stages strongly suggested in atherosclerotic plaque formation. Vascular proliferation reduces intimal thickening in arteries where the development of atherosclerosis occurs. Controlling vascular proliferation by regulating cell cycle progression is a new therapeutic strategy for atherosclerosis [17]. Many growth factors have been shown to function as mitogens for VSMC. One of them is PDGF-BB. PDGF-BB is important in vascular repair after cellular injury. It has been implicated in neointima formation and inhibitors of PDGF-BB signal transduction have been shown to decrease the formation [18]. PDGF binds to its receptor PDGFR which subsequently activate signal transduction, for example, through the PI3K pathway, and lead to downstream regulation of gene expression and the cell cycle. PDGF-BB is also found to trigger the production of extracellular matrix (ECM) and secretion of cytokines which lead to structural changes of the media and allow the VSMC to migrate from media to the inflammatory site [19]. Our study involved the use of PDGF-BB to induce VSMC proliferation and migration, and the subsequent inhibition of these processes by SR10 was investigated. PDGF-BB induced VSMC proliferation which was suppressed by the addition of SR10 (Figure 3). The suppression was not due to cytotoxicity of SR10 because SR10 alone did not decrease cell viability when compared with negative control. Result of ^3^H-thymidine uptake assay also suggested that SR10 decreased DNA synthesis which is an indication of cell proliferation (Figure 4).

The anti-proliferation of VSMC by SR10 was further confirmed by cell cycle analysis. Cells are activated by entering from the quiescent (G_0_) stage to G_1 _phase. To begin the DNA replication, the cell enters S phase for synthesis and then G_2_/M phase for mitosis. Our results indicated that PDGF-BB arrested the cells at S phase and G_2_/M phase. This means that PDGF-BB activated cell proliferation (Figure 5A). The results demonstrated that SR10 suppressed PDGF-BB-induced VSMC proliferation by decreasing cell cycle arrest at S and G_2_/M phase. Besides cell populations in different phases, regulation on different cyclin-CDK complexes was also studied. Cyclin D-CDK4 and cyclin E-CDK2 complexes regulate G_1 _and S phases transition while cyclin A-CDK2 and cyclin B-CDK1 regulate G2/M phase transition [20]. The results of Western blot showed that PDGF-BB-induced expression of cyclin D1 was suppressed by co-treatment of SR10 in a dose-dependent manner (Figure 5B). The decrease in G_1 _and S phases transition consolidated the result that SR10 inhibited PDGF-BB-induced S and G_2_/M phase cell cycle arrest, and hence inhibited VSMC proliferation. The signaling pathway of PDGF-BB-induced mitogenesis involved the activation of ERK1/2 which was shown in Figure 5C. In fact, it has been previously reported that baicalin (an active component from Scutelleria baicalensis) and corynoxeine (isolated from Uncaria rhynchophylla) also significantly inhibited PDGF-induced ERK1/2 activation [21,22].

Besides cell proliferation, VSMC migration from media to intimal space is also important in the development of atheroma. In our study, a transwell migration assay was applied to investigate the inhibitory effect of SR10 on VSMC migration. SR10 was shown to be inhibitory for VSMC migration in a dose-dependent manner (Figure 6).

Actually, VSMC proliferation and migration are two independent processes. SR10 was found to be effective in inhibiting both processes, implying that it is a potential inhibitor of atherosclerosis. This is a preliminary study using *in vitro *models. Different concentrations of SR10 were used in different experiments because different cellular models and modes of action are involved. For example, red blood cells were used to measure the inhibitory effects of SR10 on free radical-induced hemolysis while A7r5 is the smooth muscle cell line used for the measurement of proliferation and migration. In future, *in vivo *model should be applied to consolidate the observations. A concentration range of 1.25 - 5 mg/ml of SR10 was used to treat vascular smooth muscle cell *in vitro*. *In vivo *experiment will be required to test if same concentration range in mg/ml of SR10 can be reached in the host's body.

SR10 is a novel formulation and no previous study has been done for the effects of SR10 on anti-atherosclerosis. In addition, as SR10 is comprised of three herbs, the components in these herbs may have their own interactions. Therefore, it is not easy to predict the physiological effects and the bioavailable concentration of SR10 in human or animals. Future *in vivo *experiments must be done in order to assess the efficacy of SR10 in atherosclerotic treatment.

## Conclusions

This study demonstrated that SR10, a herbal mixture of *Radix Astragali, Radix Codonopsis *and *Cortex Lycii*, exhibited antioxidant activities which was effective in inhibiting red blood cell hemolysis and prolonging LDL oxidation. The anti-atherogenic effects of SR10 was also indicated by the attenuation of PDGF-induced VSMC proliferation and migration. The results of this study implied the potential application of SR10 in treating atherosclerosis.

## Declaration of competing interests

The authors declare that they have no competing interests.

## Authors' contributions

JC and JK were responsible for performing the experiments, analyzing data and drafting the manuscript. PL, CC and KF supervised the whole study and revised the manuscript. All authors have read and approved the final manuscript.

## Pre-publication history

The pre-publication history for this paper can be accessed here:

http://www.biomedcentral.com/1472-6882/11/32/prepub
